# A Text-Book of Anatomy

**Published:** 1899-07

**Authors:** 


### Book Reviews.

A TEXT-BOOK OF ANATOMY. By American Authors. Edited by FREDERIC HENRY GERRISH, M. D., Professor of Anatomy in the Medical School of Maine, Bowdoin College. Octavo, 915 pages, with 950 engravings in black and colors. Philadelphia and New York: Lea Brothers & Co. 1899.

It does seem at last as if the days of moth-eaten tradition had gone by so far as the writing of text-books on anatomy is concerned, now that Dr. Gerrish has completed his work and it has been placed before a wearied and hopeful profession. The day may come when someone will improve on Gerrish's method of writing anatomy. If it does, all well and good, but it will have to be a particularly fine bit of work.

Instruction in anatomy has always been more or less of a parrot-like job. Bones were handled, held up before the eyes of expectant freshmen, and described in the same language used in whatever text-book happened to be in use in the particular institution where anatomy was being taught. Muscular attachments were spoken of with great rapidity and an easy familiarity, a bloodvessel or two mentioned, a nerve gathered in in passing, and function spoken of as if it grew on trees,—and that was teaching anatomy. If Gerrish is used generally, as it should be, all that will be changed. There will be a methodicity in teaching and an interest which will make anatomy anything but a grind. Any man with an excellent memory can teach anatomy as it has been taught. It will take brains to teach it as Gerrish lays it down, and this fact will undoubtedly necessitate changes in the personnel of the teaching squads of some of the medical colleges in this country, all of which would be better for the colleges and the students. Students who pay fees for instruction are paying for the very best that can be given. A medical college which does not give them everything at its command is sailing under false colors and obtaining money under false pretenses--exactly the same as a professor who is supposed to teach a certain branch and goes only so far as theory permits him, stopping short of giving actual, practical experience to his students; that, too, would be sailing under false colors and obtaining money under false pretenses. The best a medical college can do nowadays in teaching anatomy is to teach it according to Gerrish. There is a man with ideas—a man who has taken a musty, tradition-gnawed subject and made it bright and interesting; a man who makes music of the rattle of dry bones; a man who puts the pink of life in the flabby flesh of death. Dr. Gerrish has had the assistance of the foremost anatomical teachers in America in the preparation of the book, which, by the way, is not at all cumbersome, as one would imagine after stuyding Gray or Morris. Dr. Gerrish, himself, has written a marvelously entertaining and instructive introductory chapter, giving definitions, systems, organs, order of topics and methods of study; he has contributed also the chapters on elementary tissues, the muscles, the fasciæ, the heart, the lymphatic system, the cerebro-spinal axis, the tongue, the ear, the organs of digestion, the organs of respiration, the urinary system, the ductless glands, relational anatomy and practical anatomy.

James Playfair McMurrich, professor of anatomy in the University of Michigan, has written on embryology; George Woolsey, professor of anatomy and clinical surgery in the Cornell University Medical College, has written on the bones, the articulations, the veins; Arthur Dean Bevan, professor of anatomy in the Rush Medical College, has written of the arteries; William Keiller, professor of anatomy in the medical department of the University of Texas, has written of the nerves, the skin, the nose, the eye; George Davis Stewart, professor of anatomy in the University and Bellevue Hospital Medical College, has written on the organs of generation.

These men are all teachers of anatomy and they all saw the evils of the systems in vogue, hence wrought changes in their teachings. What, then, was more natural than that they should draw together and by their combined labors bring forth a volume which is beyond criticism and comes like a breath of fresh air in a noisesome tomb! They avoid the unimportant, and have dealt only with those facts of human anatomy which are essential preliminaries to a rational, practical and intelligent study of physiology, surgery and internal medicine.

They have made a book which very ably takes the place of the lecture; and in the preface Dr. Gerrish has struck the key-note of the evil of modern medical education when he says : They (the authors) certainly, as a matter of policy, have admitted many things which, in their judgment, are not essential to the student, except for the aid they afford him in meeting certain conventional demands, especially on the part of examiners who may not have emancipated themselves from the trammels of tradition."

Greater stress than usual has been laid upon visceral structure, and the same may be said of surface anatomy. The chief charm of the book, aside from the brilliancy of its arrangement and construction, is the illustrating.

The illustrations, drawings and diagrams are unusually well done, remarkably free from the old-fashioned cut-and-dried appearance, and exceptionally intelligent in detail. The system of direct labelling has been adopted with great apparent improvement in the work. Summing up the work is easy: there is nothing with which to find fault, everything to praise. Truly, the medical profession should welcome the work as the most remarkable and most valuable volume of the year.

In its production Messrs. Lea Brothers & Co. have realised its genuine worth and have given to it all the art and attention to detail in medical-book producing for which they are justly famous.

